# *Bordetella holmesii* Lipopolysaccharide Hide and Seek Game with Pertussis: Structural Analysis of the O-Specific Polysaccharide and the Core Oligosaccharide of the Type Strain ATCC 51541

**DOI:** 10.3390/ijms21176433

**Published:** 2020-09-03

**Authors:** Karolina Ucieklak, Sabina Koj, Tomasz Niedziela

**Affiliations:** Hirszfeld Institute of Immunology and Experimental Therapy, 53-114 Wroclaw, Poland; karolina.ucieklak@hirszfeld.pl (K.U.); sabina.koj@hirszfeld.pl (S.K.)

**Keywords:** whooping cough, pertussis, *Bordetella holmesii*, *Bordetella pertussis*, lipopolysaccharide, O-antigen, core, NMR spectroscopy

## Abstract

Whooping cough is a highly contagious disease caused predominantly by *Bordetella pertussis*, but it also comprises of a pertussis-like illness caused by *B. holmesii*. The virulence factors of *B. holmesii* and their role in the pathogenesis remain unknown. Lipopolysaccharide is the main surface antigen of all *Bordetellae*. Data on the structural features of the lipopolysaccharide (LPS) of *B. holmesii* are scarce. The poly- and oligosaccharide components released by mild acidic hydrolysis of the LPS were separated and investigated by ^1^H and ^13^C NMR spectroscopy, mass spectrometry, and chemical methods. The structures of the O-specific polysaccharide and the core oligosaccharide of *B. holmesii* ATCC 51541 have been identified for the first time. The novel pentasaccharide repeating unit of the *B. holmesii* O-specific polysaccharide has the following structure: {→2)-α-l-Rha*p*-(1→6)-α-d-Glc*p*-(1→4)-[β-d-Glc*p*NAc-(1→3]-α-d-Gal*p*-(1→3)-α-d-Glc*p*NAc-(1→}_n_. The SDS-PAGE and serological cross-reactivities of the *B. holmesii* LPS suggested the similarity between the core oligosaccharides of *B. holmesii* ATCC 51541 and *B. pertussis* strain 606. The main oligosaccharide fraction contained a nonasaccharide. The comparative analysis of the NMR spectra of *B. holmesii* core oligosaccharide fraction with this of the *B. pertussis* strain 606 indicated that the investigated core oligosaccharides were identical.

## 1. Introduction

Bacterial genus *Bordetella* belongs to the *Alcaligenceae* family. *Bordetellae* are Gram-negative, aerobic, and typically small coccobacilli [1]. Extensive molecular analysis of the ribosomal 16S RNA extended the list of known *Bordetella* species. Currently the Genus comprises sixteen species: *B. pertussis*, *B. parapertussis*, *B. bronchiseptica*, *B. holmesii*, *B. petrii*, *B. hinzii*, *B. pseudohinzii*, *B. trematum*, *B. avium*, *B. ansorpii*, *B. flabillis*, *B. bronchialis*, *B. sputigena*, *B. muralis*, *B. tumulicola*, *B. tumbae* [2]. The most prominent for the genus *Bordetella* is *Bordetella pertussis*—exclusively human pathogen, responsible for whooping cough—a highly contagious disease of respiratory tract, especially dangerous for infants and young children. Some milder form of pertussis may be also caused by *Bordetella holmesii,* another *Bordetella*—a human pathogen predominantly isolated from immunocompromised patients [3,4]. *B. pertussis*, *B. parapertussis*, *B. bronchiseptica,* that is the classical *Bordetellae,* are closely related pathogenic bacteria and termed the “*Bordetella bronchiseptica* cluster” [5]. The initial 16 rRNA analysis of *B. holmesii* indicated that this bacterium was closely related to *B. pertussis* [1,3], but recent genomic data has suggested that there are substantial differences and thus *B. holmesii* does not conform to the classical *Bordetellae* [6]. *B. holmesii* was initially associated with the infection of the immunosuppressed post-splenectomy patients [3] and only a few years later it was isolated from patients, who manifested pertussis-like symptoms [4]. *B. holmesii* has also been reported as a causative agent of bacteremia [7], a feature in contrast to *B. pertussis*. *B. holmesii* is not a typical mammalian *Bordetella*—it is closely related to *B. pertussis* and *B. bronchiseptica* and shares ~66% of the genes with these bacteria. However, the genome content has more features resembling these of *B. avium* and *B. petrii*. Its evolutionary history can be traced back to avian *Bordetellae* [8]. Analysis of the clinical isolates of *B. holmesii* indicates that 400 genes are unique for this bacterium and were not found among other *Bordetellae*. Interestingly, each strain bears between 24 and 114 distinctive genes, and one of those genes was also detected in the *E. coli* genome. It suggests a susceptibility of *B. holmesii* to a horizontal gene transfer that could explain the acquisition of pertussis-like properties from *B. pertussis* via such transfer [9]. A limited genetic variability among different strains *B. holmesii* might indicate a fairly recent adaptation to human hosts [6]. The relation between *B. pertussis* and *B. holmesii* infections remains unknown, but cases of the co-infection have been reported recently [10,11,12]. Although the infection rate with *B. holmesii* is low, ranging initially from < 1% of the diagnosed cases in the US and Canada [11], up to 20% in France [13] and 29% in Ohio, USA of patients tested for pertussis [12]. All these data confirmed that *B. holmesii* infects adolescents and adults, however lack of specific diagnostic tools capable of detecting and differentiating *Bordetellae*, may lead to underestimation of the actual infection rates. It is vital to distinguish *B. pertussis* and *B. holmesii* infections efficiently as failing to do so may substantially underrate the efficiency of anti-pertussis vaccination. All *Bordetellae* have lipopolysaccharides as the most abundant surface antigen and an integral component of the outer membrane of the cell envelope. The structure of lipopolysaccharides (LPS) among *Bordetellae* conforms to an overall scheme of this amphiphilic molecule composed of lipid A, core oligosaccharide, and O-specific polysaccharide, but differs structurally between species and strains. To further complicate the analysis of the structural features of these LPS some additional oligosaccharide linkers between the core and the O-PS have been identified in *B. bronchiseptica* and *B. parapertussis* [14]. All these segments are vital for the physical properties of LPS and the host-pathogen interactions. *B. holmesii* is a Gram-negative, rod-shaped, slowly-growing bacterium. Since the first report on *B. holmesii* there were attempts to identify interrelation of this bacterium with *B. pertussis*, regarding the main virulence factors and antigens. As demonstrated by Zhang et al. neither DTwP nor DTaP pertussis vaccines provide cross-protection against *B. holmesii* [15]. This observation and the genome data indicate that *B. holmesii* shares some features with other *Bordetellae*, yet also differs substantially. *B. holmesii* does not produce the main protein antigens typical for *B. pertussis* and implicated in the pathogenesis of whooping-cough such as pertussis-toxin, filamentous hemagglutinin, pertactin, adenylate cyclase toxin and fimbrial proteins [8,15]. Among the virulence factors of *B. bronchiseptica* and *B. pertussis* is LPS [16]. The virulence factors of *B. holmesii* and their role in the pathogenesis remain unknown. However, data on the structural features of the LPS of *B. holmesii* are scarce. To date, only the structural analysis on the *B. holmesii* lipid A was published [16]. Thus, for the first time, we describe the structural details of *B. holmesii* strain ATCC 51541 O-specific polysaccharide and core oligosaccharide segments of LPS and confront them with these typical LPS of the “*B. bronchiseptica* cluster.”

## 2. Results

### 2.1. Isolation of Lipopolysaccharides and O-Specific Polysaccharides 

*B. holmesii* ATCC 51541 and three classical *Bordetella* species (*B. pertussis* strains 186 and 606, *B. parapertussis* strain 529 and *B. bronchiseptica* strains 530 and 1943) were cultured on the standard Stainer–Scholte (SS) medium. The structure of LPS from bacteria belonging to the “*B. bronchiseptica cluster*” are well described in the literature [14,17,18,19,20], therefore *B. pertussis, B. parapertussis*, and *B. bronchiseptica* strains were used as controls in electrophoretic and serological analysis. The growth of *B. holmesii* on the standard SS medium was weak, with very low efficiency, therefore the composition of the medium was modified. Recently published research has shown that the addition of riboflavin (10 µg/mL) in case of low *B. pertussis* density stimulated its growth [21], and likewise, we also observed a better growth of *B. holmesii*. When compared to the standard SS medium the yield of *B. holmesii* growth on SS medium with riboflavin under the same conditions was doubled (In 250 mL of medium an increase from 0.1 g to 0.2 g of dried bacterial mass was observed).

**Figure 1:**
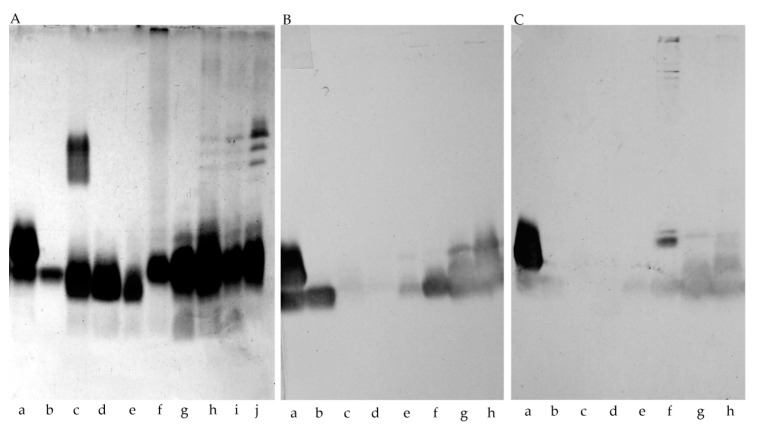
Electrophoretic profiles (**A**) of the LPS from (a) *B. pertussis* 186, (b) *Bordetella pertussis* 606, (c) *B. parapertussis* 529, (d) *B. bronchiseptica* 1943, (e) *B. bronchiseptica* 530 and different preparation of LPS *B. holmesii* ATCC 51541 (f) Bhol_prep1, (g) Bhol_prep2 and (h) water phase, (i) interphase, (j) phenol phase Bhol_prep3, Immunoblottings with rabbit polyclonal sera: anti-OS-PT (**B**), anti- complete bacterial cells of a mixture *B. pertussis* (**C**).

The LPS of *Bordetella* species were extracted from dried bacterial mass by the modified hot phenol/water method [22] and purified by ultracentrifugation [23]. The SDS-PAGE analysis of the LPS preparation of different *Bordetella* species showed the smooth-type LPS of *B. holmesii* ATCC 51541 and *B. parapertussis* 529. The rough-type LOS were detected for *B. pertussis* strains 186 and 606 as well as for *B. bronchiseptica* strains 530 and 1943 (Figure 1A). The electrophoretic separation of complete lipooligosaccharide (LOS) of *B. pertussis* yields two bands described as the “A-band” (slow-migrating) and the “B-band” (fast-migrating). The A-band is a complete LOS structure consisting of lipid A, core oligosaccharide, and the distal trisaccharide [α-d-Glc*p*NAc-(1→4)-β-d-Man*p*2NAc3NAcA-(1→3)-β-l-Fuc*p*2NAc4NMe-(1→]. B-band is an incomplete LOS that lacks the terminal trisaccharide. The intact oligosaccharide molecule is a dodecasaccharide, and OS that lacks distal trisaccharide is a nonasaccharide. In the SDS–PAGE of *B. pertussis* 186 LOS both bands are observed. In the SDS-PAGE of LOS of *B. pertussis* 606 only B-band is present, which confirms that *B. pertussis* 606 is a rough strain, producing LOS that lacks the distal trisaccharide. *B. holmesii* ATCC 51541 showed a smooth-type LPS in the SDS-PAGE analysis. We observed a band in the region of lipid A linked to the core and a set of bands in the O-antigen region. In the immunoblotting analysis, the fast migrating core-lipid A band of *B. holmesii* LPS cross-reacted with rabbit sera containing antibodies directed against complete bacterial cells of a mixture of *B. pertussis* strains 186/576/606/629 (Figure 1C) as well as with rabbit sera containing polyclonal antibodies directed against *B. pertussis* 186 OS-PT glycoconjugate (Figure 1B). Electrophoretic and serological analyses have indicated that the OS structure of *B. holmesii* core may be similar to the structure of *B. pertussis* 606 OS.

**Figure 2:**
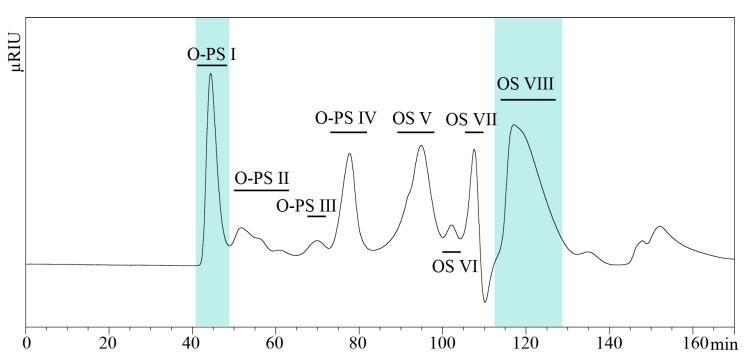
Separation of the O-PS and OS components of *B. holmesii* ATCC 51541 LPS hydrolyzate by gel-filtration chromatography on the Superdex 30 pg, with RI detector.

The heteropolysaccharide components of the LPS were released by mild acid hydrolysis and isolated by gel filtration on HiLoad 16/600 Superdex 30 prep grade column in 0.05 M acetic acid (Figure 2). All fractions were analyzed by matrix-assisted laser desorption ionization time-of-flight (MALDI-TOF) mass spectrometry and NMR spectroscopy. The fraction with the shortest retention time (Rt 40–48 min) was identified as O-specific polysaccharide (O-PS I, yield 8.3%) with the highest degree of polymerization. The largest fraction with the longest retention time (OS VIII, yield 23.3%) was recognized as the core OS. The core oligosaccharides were compared with the known *Bordetella* cores. The comparative OS analyses were performed with fraction OS VIII (Rt 110–130 min). Fractions O-PS II and O-PS III were identified as the shorter O-specific polysaccharide and fractions OS- IV–OS-VII contained complex mixtures of O-PS and OS components.

### 2.2. NMR Spectroscopy and Chemical Analysis of the O-PS I

**Figure 3:**
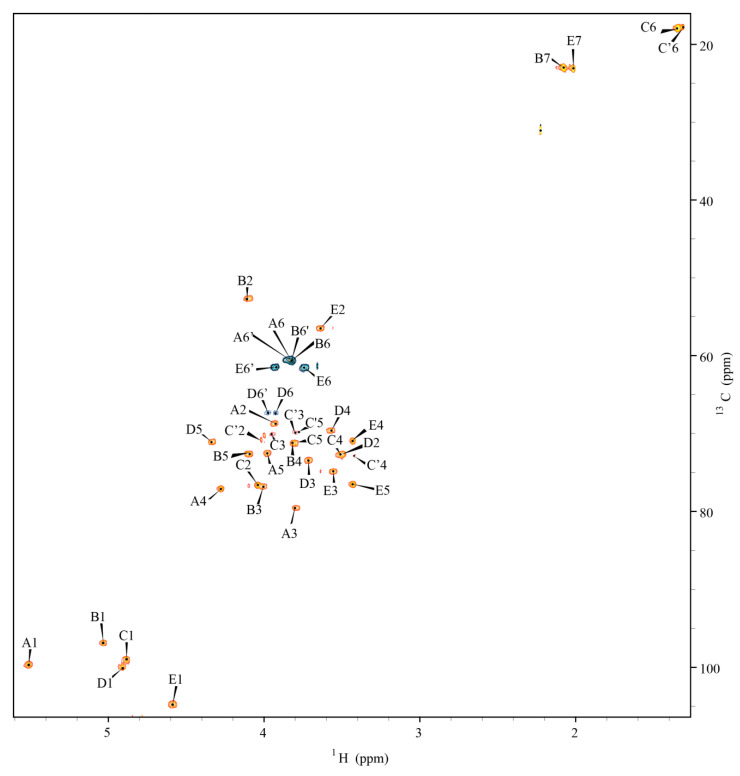
^1^H, ^13^C HSQC-DEPT spectrum of the O-PS I of *B. holmesii* ATCC 51541. The capital letters refer to carbohydrate residues as shown on the structure, and the numbers refer to protons in respective residues.

The ^1^H NMR spectrum of the isolated O-PSI indicated five anomeric signals, two acetyl resonances, and a methyl group. As the ^1^H NMR spectrum was complex, the spin systems were identified and assigned by several two-dimensional experiments, including ^1^H, ^1^H COSY; ^1^H, ^1^H TOCSY; ^1^H, ^13^C HSQC-DEPT; ^1^H, ^13^C HMBC; ^1^H, ^1^H NOESY, and ^1^H, ^13^C HSQC-TOCSY (Table 1). In ^1^H, ^13^C HSQC-DEPT spectrum (Figure 3) five sugar residues were identified based on the number of anomeric proton and carbon signals. The sugar residues are denoted with the uppercase letters through the manuscript. 

**Figure 4:**
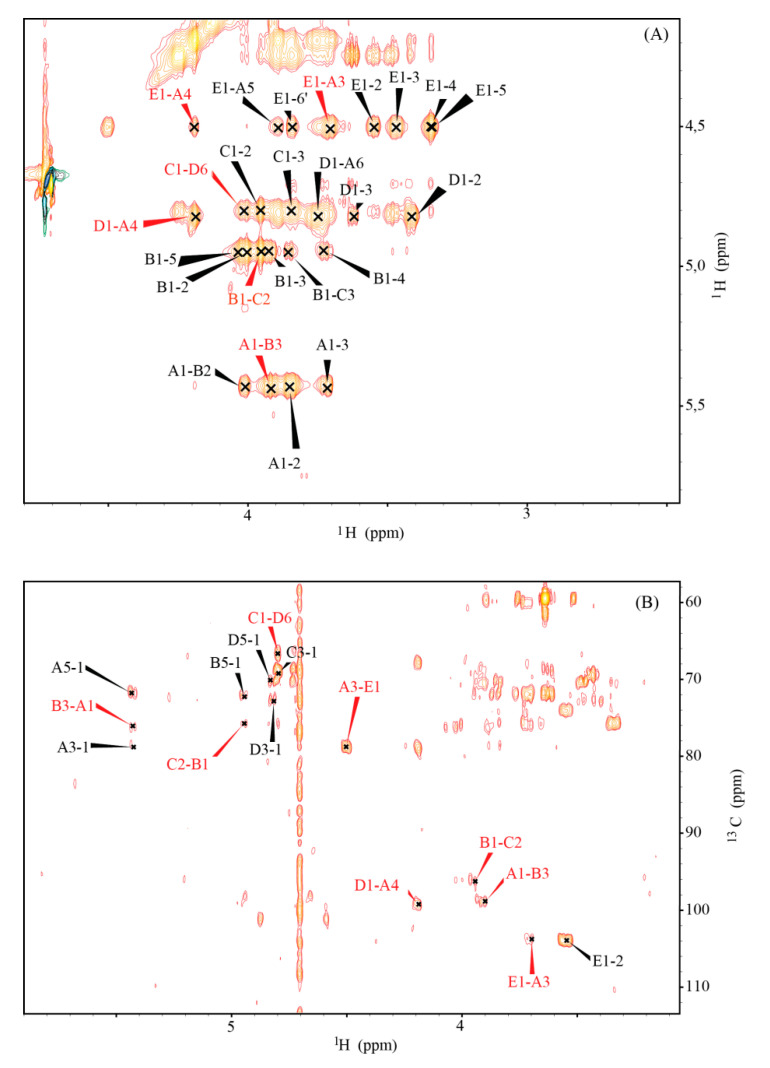
Part of the ^1^H, ^1^H NOESY spectrum (**A**) and selected ^3^J_H,C_—connectivities in the ^1^H,^13^C HMBC **(B**) spectrum of O-PS I *B. holmesii* ATCC 51541. Inter-residue cross-peaks are marked red.

Residue **A** (δ_H1_/δ_C1_ 5.51/99.7) was identified as the 3,4-disubstituted α-d-Gal*p* [→3,4)-α- d-Gal*p*- (1→] based on the large coupling between the vicinal protons H-2 and H-3 and the low values of coupling constant between H-3, H-4, and H-5, as well as the relatively high value of the chemical shifts of the C-3 (δ_C_ 79.6 ppm) and C-4 (δ_C_ 77.1 ppm) signals. Residue **B** (δ_H1_/δ_C1_ 5.03/96.8) was characterized as the 3-substituted α-d-Glc*p*NAc [→3)-α-d-Glc*p*NAc-(1→] based on the large couplings among H-2, H-3, H-4, H-5, H-6 protons in the spin system, and the high chemical shift value of the C-3 (δ_C_ 76.8 ppm) carbon signal. The chemical shift of the C-2 signal (δ_C_ 52.7 ppm) was characteristic of the carbon atom with the amine substitution. Residue **C** (δ_H1_/δ_C1_ 4.89/99.0) was assigned as the 2-substituted α-l-Rha*p* [→2)-α-l-Rha*p*-(1→] based on the small couplings of vicinal protons H-1, H-2 and H-3 and the high values of couplings between H-3, H-4 and H-5, the high chemical shift value of the C-2 (δ_C_ 76.3 ppm) carbon signal and based on the characteristic signal of the exocyclic CH_3_ group (δ_C_ 17.9, δ_H_ 1.34 ppm). The presence of the C**′** variant with chemical shifts similar to residue **C** was attributed to a non-reducing terminal variant of residue C, defining the biological repeating unit of the *O-*specific polysaccharide. Residue **D** (δ_H1_/δ_C1_ 4.92/100.0) was characterized as the 6-substituted α-d-Glc*p* [→6)-α-d-Glc*p*-(1→] based on the large couplings between H-2, H-3, H-4, and H-5 protons in the spin system and also the high value of the C-6 (δ_C_ 67.4 ppm) carbon chemical shift. Residue **E** (δ_H1_/δ_C1_ 4.59/104.8) was identified as the terminal β-d-Glc*p*NAc [β-d-Glc*p*NAc-(1→] based on the large couplings between all protons in the spin system. The low chemical shift of the C-2 signal (δ_C_ 56.5 ppm) indicated the amine substitution. Substitutions with the N-acetyl groups were identified in an ^1^H,^13^C HMBC experiment using the correlation of the carbonyl carbon of the acetyl groups at δ_C_ 175.1 ppm and δ_C_ 175.4 ppm to H-2 protons of residues **B** (δ_H_ 4.11 ppm) and **E** (δ_H_ 3.64 ppm), respectively. The J_C1, H1_ coupling values obtained from the non-decoupled HSQC experiment confirmed the α-pyranosyl configuration of residues **A** (179 Hz), **B** (172 Hz), **C** (172 Hz) and **D** (172 Hz) and the β-pyranosyl configuration for residue **E** (164 Hz). The sequence of monosaccharides in the O-PS I was determined using inter-residue correlations observed as cross-peaks between the transglycosidic protons in the ^1^H,^1^H NOESY experiment, and between the anomeric protons and carbons at the linkage position in the ^1^H,^13^C HMBC spectrum (Table 2, Figure 4).

**Figure 5:**
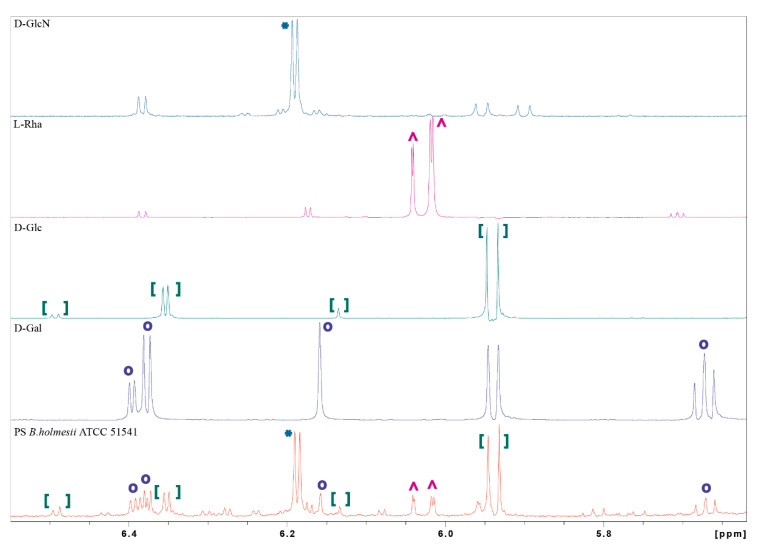
Comparison of NMR spectral profiles in the range of 5.5–6.5 ppm for O-(S)-2-methyl butyrate derivatives of monosaccharide standards and the *B. holmesii* O-PS hydrolyzate. The signs “o, *, ^, []” indicate the standard signals identified in the O-PS. The spectra were obtained for acetone-d6 solutions at 25 °C and calibrated to the internal reference of acetone-d5 (δ 2.05 ppm).

Sugar analysis of the O-PS I confirmed the presence of rhamnose, glucose, galactose, and N-acetylglucosamine. The absolute configuration of the monosaccharide components of PS *B. holmesii* was confirmed by the method described by York et al. [24]. This method allows us to define the absolute configuration of sugar residues in the PS using only NMR spectroscopy. The O-(S)-2-methyl butyrate (SMB) derivatives of d- and l- monosaccharides are diastereomeric and can be differentiated by comparing their chemical shift and coupling patterns. The polysaccharides were hydrolyzed with 2 M TFA and the resulting monosaccharides were converted into (SMB) derivatives, and then their absolute configurations were analyzed by ^1^H NMR spectroscopy (Figure 5). The samples were dissolved in deuterated acetone (acetone-d6) and all the spectra were calibrated to the internal reference of acetone-d5 (δ 2.05 ppm). Comparison of ^1^H NMR profile of the SMB-modified PS hydrolysate of *B. holmesii* ATCC 51541 and the SMB-derivatives of the selected monosaccharides confirmed the presence of d-Glc, d-Gal and l-Rha. The analysis also revealed the presence of an SMB-derivative of d-GlcN instead of the expected SMB-derivative of GlcNAc. This observation can be explained by the release of the N-acetyl group from the GlcNAc during hydrolysis in concentrated acid, and thus required the use of GlcN as an additional standard. The presence of GlcNAc in the native O-PS was supported by NMR and sugar analysis data. 

### 2.3. Structural Analysis of the O-PS I by Mass Spectrometry

**Figure 6:**
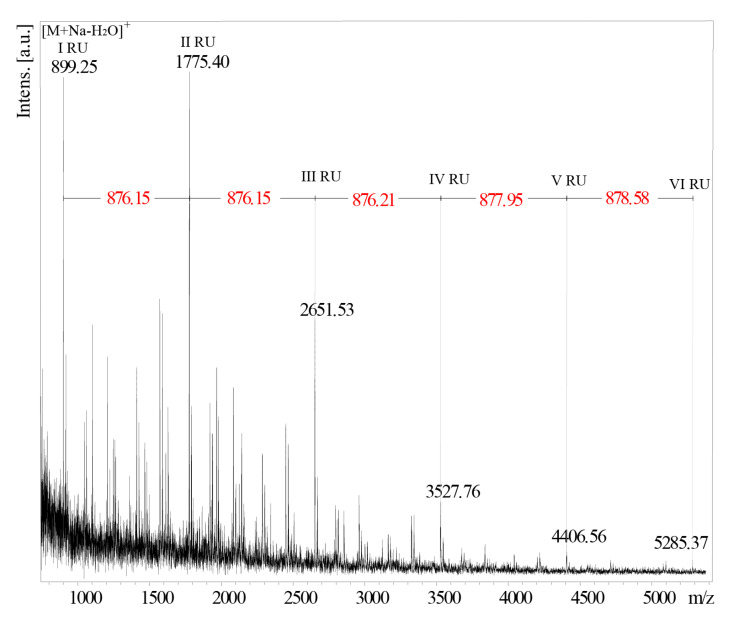
MALDI TOF-MS spectrum of oligosaccharides and trimmed O-PS segments obtained by partial acid hydrolysis of the O-PS I of *B. holmesii* ATCC 51541 (26th day of the experiment). The spectrum was obtained in the positive reflectron mode with the DHB as a matrix.

As the attempts to obtain MALDI-TOF spectra of the intact O-PS I failed, the mass of the repeating unit of *B. holmesii* strain ATCC 51541 has been deduced from the analysis of the partially hydrolyzed O-PS fractions obtained after treatment with 48% HF at −20 °C for 26 days. The MALDI-TOF mass spectrum (Figure 6), recorded in the positive polarization reflectron mode, after partial hydrolysis of PS *B. holmesii*, contained main ions at *m/z* 899.25, *m/z* 1775.40, *m/*z 2651.53, *m/z* 3527.63, *m/z* 4406.56 and *m/z* 5285.37 with a mass difference of ~876 Da between the main signals. This value corresponds to the theoretical mass of the subunit devoid of the water molecule. The dominant ion of *m/z* 899.25, was interpreted as the sodium adduct of a single repeating unit devoid of a water molecule [M+Na-H_2_O]^+^. These results confirm the theoretical mass (876.32 Da) of the O-PS repeating unit of *B. holmesii*.

**Figure 7:**
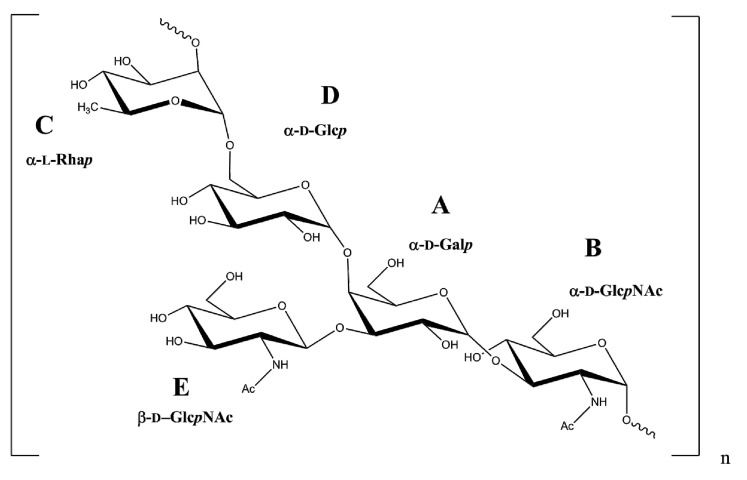
Structure of the O-repeating unit of the O-PS of *B. holmesii* ATCC 51541.

The combined NMR and MS data showed that the repeating unit of the O-antigen of *B. holmesii* ATCC 51541 is the →2)-α-l-Rha*p*-(1→6)-α-d-Glcp-(1→4)-[β-d-GlcpNAc-(1→3)]-α-d-Galp-(1→3)-α-d-GlcpNAc-(1→ pentasaccharide with the following structure (Figure 7).

### 2.4. Structural Analysis of the OS Core 

**Figure 8:**
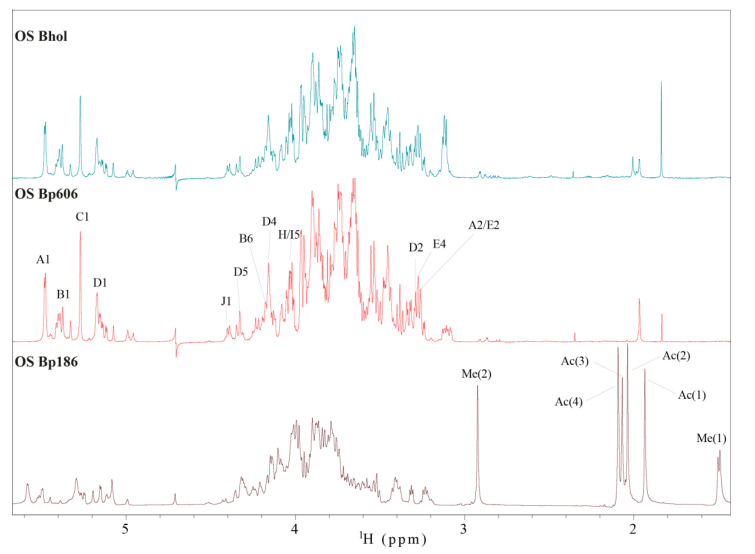
Structure reporter groups in the ^1^H NMR spectra of the oligosaccharides of *B. pertussis* (strains 186 and 606) and *B. holmesii* ATCC 51541. The capital letters refer to carbohydrate residues as shown on the structure, and the numbers refer to protons in respective residues. The resonances marked with Me(1), N-methyl, Me(2), exocyclic CH_3_ and Ac(4), N-acetyl of l-Fuc*p*2NAc4NMe, Ac(1) and Ac(3), N-acetyls of Man*p*2NAc3NAcA and Ac(2), N-acetyl of Glc*p*NAc indicate the distal [α-d-Glc*p*NAc-(1→4)-β-d-Man*p*2NAc3NAcA-(1→3)-β-l-Fuc*p*2NAc4NMe-(1→] trisaccharide in the structure of *B. pertussis* 186 LOS.

An initial SDS–PAGE analysis of the intact LPS of *B. holmesii* and the observed cross-reactivities of the anti-*B. pertussis* 186 OS-PT antibodies with the fast migrating bands suggested some structural similarity with the core oligosaccharides of *B. pertussis* strains 186 and 606 (Figure 1), thus we compared the ^1^H NMR profiles of the corresponding oligosaccharides. ^1^H NMR spectra of the *B. pertussis* strains 186 and 606 OS (Figure 8) provide information on the reporter groups in the ^1^H NMR spectra of these oligosaccharide structures. As mentioned before, *B. pertussis* produces two types of LOS. The complete OS molecule is a dodecasaccharide that includes the distal trisaccharide and the incomplete OS molecule is devoid of it. Chemical shift values for *B. pertussis* 186 have been reported [14,19] and are in line with the data published for the *B. pertussis* 1414 strain [17]. The *B. pertussis* 606 strain produces only one type of LOS, comprising lipid A linked to a nonasaccharide (Tables S1 and S2). The nonasaccharide is easily identifiable as it lacks the main reporter groups of the distal trisaccharide of *B. pertussis* LOS—the signals of the N-methyl resonance and the deoxy-group of β-l-Fuc*p*2NAc4NMe. We compared ^1^H NMR spectrum of yet unknown core OS of *B. holmesii* ATCC 51541 with the OS of *B. pertussis* strains 186 and 606. Notably, the observed similarity of the *B. holmesii* ATCC 51541 OS profile in the anomeric region in comparison with the ^1^H NMR profile of *B. pertussis* strain 606 OS indicated common structural elements.

**Figure 9:**
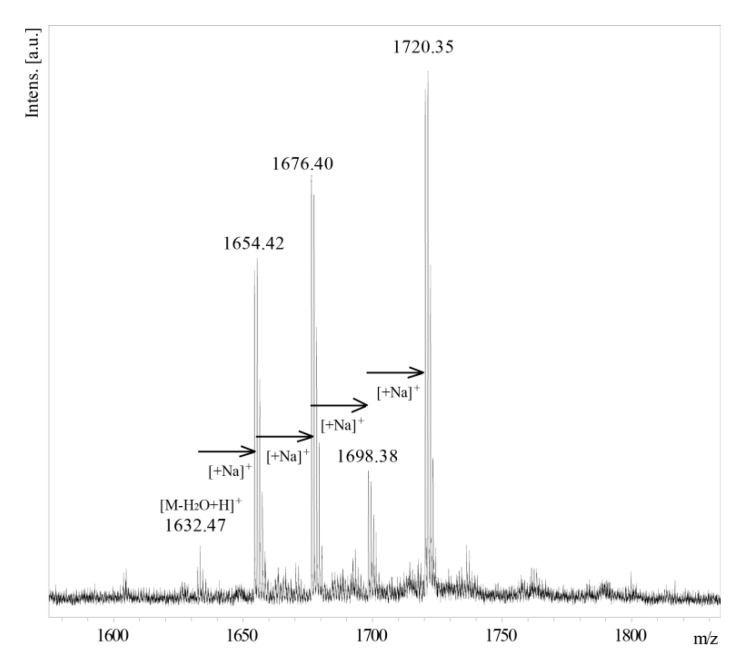
MALDI-TOF MS spectrum of OS VIII *B. holmesii* ATCC 51541 in positive reflectron mode with DHB as a matrix.

The MALDI-TOF MS spectrum (Figure 9) recorded for the OS VIII of *B. holmesii* showed a series of ions with *m/z* 1632.47, *m/z* 1654.42, *m/z* 1676.40, *m/z* 1698.38 and *m/z* 1720.35. The ion at *m/z* 1632.47 corresponds to a theoretical monoisotopic mass of nonasaccharide (1649.5 Da) devoid of a water molecule. The difference between successive ions correlates with the multiple sodium adduct. In the gel-filtration chromatography, the Rt (110–130 min) of the main OS fraction of *B. holmesii* core (OS VIII) matches up with the Rt (112–135 min) of the main OS fraction of *B. pertussis* 606. These observations were further supported by NMR data.

To verify the structural similarities between the core oligosaccharides of *B. holmesii* ATCC 51541 and *B. pertussis* 606, the corresponding spin systems of the both OS were identified and assigned by two dimensional NMR experiments, including ^1^H, ^1^H COSY; ^1^H, ^1^H TOCSY; ^1^H, ^13^C HSQC-DEPT; ^1^H, ^13^C HMBC; ^1^H, ^1^H NOESY, and ^1^H, ^13^C HSQC-TOCSY. Here we describe the chemical shift values of all individual sugar residues for the *B. holmesii* ATCC 51541 (Table 3) and *B. pertussis* 606 for the first time (Table S1). 

Residue A (δ_H1_/δ_C1_ 5.48/97.3 ppm) was identified as the 4-substituted α-Glc*p*N [→4)-α-Glc*p*N-(1→] based on large coupling constants between H-2, H-3, H-4, and H-5 in the spin system, as well as the relatively high value of the chemical shift of the C-4 (δ_C_ 74.9 ppm) signal. The chemical shift of the C-2 signal (δ_C_ 54.5 ppm) implied substitution with an amine group. Residue B (δ_H_/δ_C_ 5.39/99.7 ppm) was recognized as the 2,7-disubstituted-l-*glycero*-α-d-*manno*-Hep*p* [→2,7-l-α-d-Hep*p*-(1→] from the ^1^H and ^13^C chemical shifts, the small vicinal couplings between H-1, H-2 and H-3 and the relatively high chemical shifts of the C-2 (δ_C_ 79.8 ppm) and C-7 (δ_C_ 70.4 ppm) signals. Residue C (δ_H1_/δ_C1_ 5.27/101.2 ppm) was assigned as the terminal l-*glycero*-α-d-*manno*-Hep*p* [l-α-d-Hep*p*-(1→] based on the ^1^H and ^13^C chemical shifts and thee small vicinal couplings between H-1, H-2, and H-3. Residue **D** (δ_H1_/δ_C1_ 5.17/94.4) was identified as the terminal α-Gal*p*NA [α-Gal*p*NA-(1→] based on the large couplings between vicinal protons H-1, H-2, and H-3 and the small couplings among H-3, H-4, and H-5. The low chemical shift of the C-2 signal (δ_C_ 50.5 ppm) indicated substitution with an amine group. The characteristic five-proton spin system and the high values of the chemical shifts of H-4 (δ_H_ 4.16 ppm), H-5 (δ_H_ 4.33 ppm), and C-5 (δ_C_ 175.1 ppm) resonances allowed to identify this residue as aminouronic acid. Residue **E** (δ_H1_/δ_C1_ 5.12/96.0) was recognized as the terminal α-Glc*p*N [α-Glc*p*N-(1→] based on the large coupling constants between H-2, H-3, H-4, and H-5 in the spin system and the chemical shift of C-2 signal (δ_C1_ 54.1 ppm). Residue **F** (δ_H1_/δ_C1_ 5.07/97.8) was characterized as the 3,4-disubstituted-l-*glycero*-α-d-*manno*-Hep*p* [→3,4-l-α-d-Hep*p*-(1→] from the ^1^H and ^13^C chemical shifts, the small vicinal couplings between H-1, H-2 and H-3 and the high chemical shifts of the C-3 (δ_C_ 76.7 ppm) and C-4 (δ_C_ 71.7 ppm) signals. Residues **H** (δ_H1_/δ_C1_ 4.99/101.0 ppm) and **I** (δ_H1_/δ_C1_ 4.95/101.2 ppm) were recognized as α-Glc*p*A [α-Glc*p*A-(1→] based on the five-proton spin systems, the large chemical shifts of H-4 (δ_H_ 3.39 ppm), H-5 (δ_H_ 4.03 ppm), and C-5 (δ_C_ 176.6 ppm) signals for residue **H** and the chemical shifts of H-4 (δ_H_ 3.38 ppm), H-5 (δ_H_ 4.01 ppm), and C-5 (δ_C_ 176.7 ppm) for residue **I** as well as large couplings among H-2, H-3, H-4, and H-5. Residue **J** (δ_H1_/δ_C1_ 4.39/101.4) was assigned as the 4,6-disubstituted-β-Glc*p* [→4,6-β-Glc*p*N-(1→] based on the large couplings among all protons in the spin system and the relatively high value of the chemical shift of the C-4 (δ_C_ 79.0 ppm) and C-6 (δ_C_ 67.3 ppm) carbon signals.

As LPS hydrolysis and chromatographic separations were performed in 1.5% and 0.05 M acetic acid, respectively, therefore in 2D ^1^H and ^13^C HSQC-DEPT spectra two forms of 4,7-anhydro-3-deoxy-d-*manno*-2-octulofuranosonic acid instead of 2-keto-3-deoxy-d-*manno*-octulosonic acid (Kdo*f* and Kdo*f*’) were observed. At low pH, Kdo is unstable and is converted into different forms [25,26]. Specifically, it is an indication of the presence of phosphate at the C-4 position of Kdo in the native OS as the observed forms of 4,7-anhydro-3-deoxy-d-*manno*-2-octulofuranosonic acid are formed in the process of β-elimination of phosphate from this position [27]. In ^31^P NMR spectra of the OSVIII fraction phosphates were not detected (data not shown), but the signals of 4,7-anhKdo*f* and 4,7-anhKdo*f’* imply the presence of phosphate groups in native OS.

The J_C1, H1_ coupling values obtained from the non-decoupled HSQC experiment indicated the α-pyranosyl configuration of residues **A** (175 Hz), **B** (178 Hz), **C** (170 Hz), **D** (171 Hz), **E** (173 Hz) and **H**/**I** (170 Hz) and the β-pyranosyl configuration for residue **J** (161 Hz). The sequence of sugar residues was confirmed using inter-residue cross-peaks between the transglycosidic protons observed by ^1^H, ^1^H NOESY experiments, and between the anomeric protons and carbons at the linkage position in HMBC spectra. The complete ^1^H and ^13^C chemical shift data for the core oligosaccharides of *B. holmesii* ATCC 51541 and *B. pertussis* 606 have been included in the Supplementary Materials.

**Figure 10:**
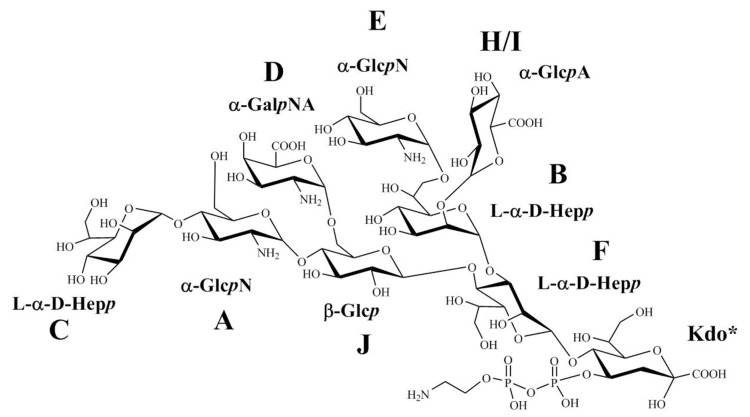
Structure of OS *B. holmesii* ATCC 51541, * Kdo was detected in the OS VIII fraction in the form of 4,7-anhydro-3-deoxy-d-manno-2-octulofuranosonic acid. The presence of ethanolamine is tentative (for indication see the supplementary data).

The results from all NMR experiments indicated that the structure of the core isolated in the main OS VIII fraction of *B. holmesii* ATCC 51541 was identical to the OS of *B. pertussis* 606 (Figure 10).

## 3. Discussion

We present here the structures of an O-specific polysaccharide, including the structure of the biological repeating unit and a core oligosaccharide of *Bordetella holmesii* ATCC 51541 lipopolysaccharide in relation to the classical *Bordetellae*. Current understanding of the whooping-cough etiology has changed, since it is not restricted to infections exclusively caused by *B. pertussis*, but embraces also pertussis-like illnesses caused by *B. holmesii* and *B. parapertussis*. *B. pertussis* is still a primary causative agent of whooping-cough, with *B. holmesii*, emerging as the second, and followed by *B. parapertussis* [10,12,13,28]. The relation between *B. pertussis* and *B. holmesii* infections remains unknown, but cases of the co-infection have been reported recently. More importantly, there is no effective vaccine against *B. holmesii,* and the existing pertussis DTwP and DTaP vaccines do not provide cross-protection in animal models of *B. holmesii* infection [15,29]. Thus, it is vital to distinguish *B. pertussis* and *B. holmesii* infections efficiently as failing to do so may substantially underestimate the efficiency of anti-pertussis vaccination. *B. holmesii* does not produce the main protein antigens typical for *B. pertussis* and implicated in the pathogenesis of whooping-cough [8,15]. At the moment further studies are required to identify and isolate virulence factors of *B. holmesii* that could become possible antigenic components of new pertussis vaccine compositions and could protect against both *B. pertussis* and *B. holmesii*.

One of the common virulence factors and the essential constituent of the bacterial membrane of all *Bordetellae* is lipopolysaccharide [16]. The lipopolysaccharides (LPS) of *Bordetellae* differ structurally between species and strains. Lipid A and core oligosaccharide moieties constitute the most conservative part of LPS across the genus. The basic structure of O-specific polysaccharides of the *B. bronchiseptica* and *B. parapertussis* were defined as homopolymers of the 1,4-linked 2,3-diacetamido-2,3-dideoxy-α-galacturonic acid, additionally modified at the non-reducing end terminal sugar by non-carbohydrate residues (e.g., formylation, substitution with aminoacids) [14]. These variable modifications define the antigenic properties of the O-antigens at the strain level. Interestingly, *B. pertussis* does not express O-antigen. Instead, it can be replaced with a single trisaccharide unit, forming two types of lipooligosaccharides (LOS)—One, built of a dodecasaccharide and another a nonasaccharide linked to lipid A. The polyclonal antibodies directed against the whole bacterial cells of *B. pertussis* (anti-mixture of *B. pertussis* strains 186/576/606/629) and these obtained against the neoglycoconjugate of the complete core OS of *B. pertussis* with pertussis toxin reacted with the fast-migrating LPS fractions of *B. holmesii*. No reaction was observed with the slow-migrating LPS fractions of *B. holmesii* cultured on standard Stainer–Scholte medium. Both SDS-PAGE analysis and the observed cross-reactions of the *B. holmesii* LPS with the polyclonal antibodies directed against the LOS-derived antigens of *B. pertussis* suggested the similarity between the core oligosaccharides of *B. holmesii* ATCC 51541 and *B. pertussis* strain 606. Moreover, this observation was in agreement with that reported by Caroff et al. [18]. The results obtained from the comparative analysis of the NMR spectra of *B. holmesii* core oligosaccharide fraction with this of the *B. pertussis* strain 606 indicated clearly that the investigated core oligosaccharides were identical. Thus the core OS of *B. holmesii* conforms to the core nonasaccharide-type that prevails among *Bordetellae* LPS. The NMR analysis of O-PSI was further supported by mass spectrometry on the partially depolymerized preparations and indicated a unique structure of the pentasaccharide O-repeats, containing one deoxy sugar (Rha*p*), one galactose (Gal*p*), one glucose (Glc*p*), and two N-acetylglucosamine (Glc*p*NAc) residues. Although the isolation of a fraction comprising a single O-repeat linked to the core OS failed, the presence of an un-substituted variant of residue **C** at the non-reducing end of the O-PS allowed to define the biological repeating unit. The herein reported O-polysaccharide and core oligosaccharide are the first ones described for the *B. holmesii* type strain ATCC 51541.

## 4. Materials and Methods 

### 4.1. Bacteria

*Bordetella holmesii* strain ATCC 51541 (DSM 13416) was obtained from DSMZ collection (Leibniz-Institut, Berlin, Germany). *B. pertussis* strains 186 and 606, used in the current wP vaccine manufactured in Poland, were acquired from the National Medicines Institute (Warsaw, Poland) [30]. *Bordetella parapertussis* PCM 529 (ATCC 15311), *Bordetella bronchiseptica* PCM 530 (ATCC 19395), and *Bordetella bronchiseptica* PCM 1943 (ATCC 4617) came from the PCM collection (Hirszfeld Institute of Immunology and Experimental Therapy, Polish Academy of Sciences, Wrocław, Poland). The strains were stored as bacterial suspensions in PBS containing 20% glycerol, at −70 °C. Bacteria were grown on charcoal agar medium supplemented with 10% defibrinated sheep blood (GRASO Biotech, Owidz, Poland) and then transferred to the liquid medium. *B. pertussis* strains were cultured using Stainer–Scholte medium at 37 °C for 72 h and *B. holmesii* was cultured in the modified Stainer–Scholte medium with the addition of riboflavin. Bacteria were killed with 1% phenol, harvested by centrifugation (4000× *g*, 30 min, 4 °C) (Sorvall Lynx 6000), suspended in water and freeze-dried. 

### 4.2. Lipopolysaccharides and O-Specific Polysaccharide Fractions

LPS was extracted from lyophilized bacterial cells by the modified hot phenol/water extraction method [22] and purified by ultracentrifugation [23]. The modified extraction included an extra step prior to the addition of phenol. Briefly, killed and lyophilized bacteria were suspended in 0.05 M phosphate buffer at pH 7.4 and lysozyme (EC 3.2.1.17, specific activity ≥ 40,000 U/mg) was added in portions (10 mg per one g of dry bacterial mass) and the suspension was incubated for 18 h at 25 °C with stirring [31]. LPS (45 mg) was hydrolyzed with 1.5% acetic acid at 100 °C for 15 min and subsequently, 12 mg of water-soluble heteropolysaccharide was isolated. The supernatant was fractionated using the semi-preparative HPLC UltiMate 3000 chromatographic system (Dionex Corporation, Sunnyvale, CA, USA) on HiLoad 16/600 Superdex 30 prep grade column (30 mm × 124 cm, grain size 34 μm, GE Healthcare, Chicago, IL, USA) equilibrated with 0.05 M acetic acid. The chromatography yielded the main fraction containing O-specific polysaccharide (O-PS I, ~1 mg), separated from shorter O-specific polysaccharide chains (O-PS II, 0.5 mg, O-PS III, 0.25 mg, O-PS IV, 1 mg), the core substituted with phosphates (OS V-OS VII, <0.1 mg) and main OS fraction containing unsubstituted core oligosaccharides (OS VIII, 2.8 mg). Eluates were monitored with a Shodex RI-102 detector (Showa-Denko, Tokyo, Japan). All fractions were checked by NMR spectroscopy and matrix-assisted laser desorption/ionization time-of-flight (MALDI-TOF) mass spectrometry (MS).

### 4.3. Analytical Procedures

Monosaccharides were analyzed as their alditol acetates by GC-MS using a Thermo ITQ™ 1100 GC-Ion Trap mass spectrometer coupled with a Trace™ 1310 gas chromatograph (Thermo Scientific™) equipped with HP-5M S column (30 m, ID = 0.25 mm, dF = 0.25 μm) (Agilent Technologies, Lexington, MA, USA) and a temperature gradient 150–270 °C at 8 °C·min^−1^. The absolute configurations of the sugars were determined using ^1^H NMR spectroscopy by converting the polysaccharide hydrolysate components and relevant monosaccharide standards into O-(S)-2-methyl butyrate derivatives as described by York et al. [24]. For NMR analyzes, the samples were dissolved in acetone-d6 (~160 μL) and transferred to 3 mm diameter NMR tubes.

### 4.4. Partial HF Hydrolysis 

The polysaccharide O-PS I fraction (0.2 mg, 200 μL) was hydrolyzed with 48% hydrofluoric acid at −20 °C. The O-PS I was dissolved in HF solution portioned and stored at −20 °C. Everyday a sample (10 μL) was taken and the progress of hydrolysis was checked by MALDI-TOF MS. The hydrolysis was ended after 30 days when no polymerized material was detected.

### 4.5. SDS-PAGE and Serological Analysis

The LPS was analyzed by SDS-PAGE according to the method of Laemmli [32]. The LPS bands were visualized by the silver staining method [33] and by immunoblotting using polyclonal rabbit antisera specific for the whole cell of *B. pertussis* (The serum was obtained by immunization with a mixture of *B. pertussis* strains 186/576/606/629) and for the core oligosaccharide of *B. pertussis* 186 (OS-PT) and the LOS-derived pentasaccharide (penta-PT) in separate experiments. OS-PT and penta-PT conjugates and the corresponding sera were described in the US (US 9878051 B2) patent [34]. All polyclonal rabbit sera were from our Laboratory collection. Immunoblotting was done as previously described [23]. The detection systems consisted of a goat anti-rabbit IgG conjugated with alkaline phosphatase (Bio-Rad, Hercules, CA, USA) as a second antibody and 5-bromo-4-chloro-3-indolyl phosphate-nitroblue tetrazolium.

### 4.6. Mass Spectrometry

MALDI-TOF MS spectra of polysaccharides, oligosaccharides, and lipid A were acquired using UltrafleXtreme (Bruker Daltonik GmbH, Bremen, Germany) with a time-of-flight detector. Spectra were recorded in positive and negative modes. 2,5-Dihydroxybenzoic acid was used as a matrix.

### 4.7. NMR Spectroscopy

NMR spectra of the isolated polysaccharide and oligosaccharides were recorded for ^2^H_2_O solutions at 25 °C on Bruker Avance III 600 MHz spectrometer (Bruker Biospin GmbH, Rheinstetten, Germany) using 5 mm QCI cryprobe. 3 mm tubes (~160 μL) were used for the measurements. Polysaccharide and oligosaccharide fractions were repeatedly exchanged with ^2^H_2_O with intermediate lyophilization. Acetone (δ_H_/δ_C_ 2.225/31.05 ppm) was used as an internal reference. The data were acquired and processed using TopSpin software (Bruker BioSpin GmbH, Rheinstetten, Germany). The processed spectra were assigned with the help of the NMRFAM-SPARKY program [35]. The signals were assigned by one- and two-dimensional experiments (COSY, TOCSY, NOESY, HMBC, HSQC-DEPT, and HSQC-TOCSY). In the TOCSY experiments, the mixing times used were 30, 60, and 100 ms. The coupling patterns within the identified spin-systems in the 2D TOCSY experiments facilitated the identification of individual monosaccharide residues. The delay time in the HMBC experiment was 60 ms and the mixing time in the NOESY experiment was 100 ms. 

## 5. Conclusions

The relation between *B. pertussis* and *B. holmesii* infections remains unknown. To date there is no effective vaccine against *B. holmesii,* and the existing pertussis DTwP and DTaP vaccines do not provide cross-protection in animal models of *B. holmesii* infection [15,29]. Lipopolysaccharide is one of the common virulence factors and the essential constituent of the bacterial membrane of all *Bordetellae* [16]. In the current research we have elucidated the structures of the O-specific polysaccharide and the core oligosaccharide of *B. holmesii* ATCC 51541: (1) The *B. holmesii* O-specific polysaccharide has been identified as the novel pentasaccharide biological repeating unit (Figure 7). (2) The comparative analysis of the NMR spectra of *B. holmesii* core oligosaccharide fraction with this of the *B. pertussis* strain 606 indicated their structural identity. These observations were in agreement with SDS-PAGE analysis and the detected cross-reactions of the *B. holmesii* ATCC 51541 LPS with the polyclonal antibodies directed against the LOS-derived antigens of *B. pertussis.* Therefore, the core nonasaccharide-type, prevailing among *Bordetellae* LPS could facilitate a design of a cross-protective neoglycoconjugate as a potential vaccine component.

## Figures and Tables

**Table 1:** ^1^H and ^13^C NMR chemical shifts of the O-PS of *B. holmesii* strain ATCC 51541 ^a^.

Residue	Chemical Shifts (ppm)					
	H-1	H-2	H-3	H-4	H-5	H-6, H-6′
	C-1	C-2	C-3	C-4	C-5	C-6
**A** →3,4)-α-d-Gal*p*-(1→	5.51	3.93	3.80	4.28	3.98	3.82, 3.82
	99.7	68.7	79.6	77.1	72.5	60.7
**B** →3)-α-d-Glc*p*NAc-(1→	5.03	4.11	4.01	3.82	4.09	3.78, 3.78
	96.8	52.7	76.8	71.2	72.6	60.7
**C** →2)-α-l-Rha*p*-(1→	4.89	4.04	3.95	3.51	3.79	1.34
	99.0	76.3	70.1	72.7	71.2	17.9
**C′** α-l-Rha*p*-(1→ ^b^	4.89 ^c^	4.02	3.81	3.44	3.78	1.31
	99.0	70.8	69.8	73.0	69.8	17.8
**D** →6)-α-d-Glc*p*-(1→	4.92	3.51	3.72	3.57	4.34	3.93, 3.97
	100.0	72.7	73.5	69.7	71.1	67.4
**E** β-d-Glc*p*NAc-(1→	4.59	3.64	3.56	3.43	3.43	3.74, 3.82
	104.8	56.5	74.9	71.0	76.5	61.5

^a^ Spectra were obtained for ^2^H_2_O solutions at 25 °C. Acetone (δ_H_/δ_C_ 2.225/31.05 ppm) was used as an internal reference; ^b^ the non-reducing terminal variant of residue **C**. ^c^ δ_H1_/δ_C1_ signals of the residues **C** and C’ were not resolved.

**Table 2:** Selected inter-residue NOE and ^3^J_H_, _C_-connectivities from the anomeric atoms of the *O-*antigen repeating unit of PS *B. holmesii* ATCC 51541.

Residue	Atom H-1/C-1	Connectivities to		Inter-Residue
	(ppm)	δ_C_	δ_H_	Atom/Residue
**A** →3,4)-α-d-Gal*p*-(1→	5.51/99.7	76.8	4.01	C-3, H-3 of **B**
**B** →3)-α-d-Glc*p*NAc-(1→	5.03/96.8	76.3	4.04	C-2, H-2 of **C**
**C** →2)-α-l-Rha*p*-(1→	4.89/99.0	67.4	3.93/3.97	C-6, H-6/6′ of **D**
**D** →6)-α-d-Glc*p*-(1→	4.92/100.0	77.1	4.28	C-4, H-4 of **A**
**E** β-d-Glc*p*NAc-(1→	4.59/104.8	79.6	3.80	C-3, H-3 of **A**

**Table 3:** ^1^H and ^13^C NMR chemical shifts of the *B. holmesii* ATCC 51541 core oligosaccharide ^a^.

Residue	Chemical Shifts (ppm)							
	H-1	H-2	H-3	H-4	H-5	H-6, H-6′	H-7	H-8, H-8′
	C-1	C-2	C-3	C-4	C-5	C-6	C-7	C-8
**Kdo** 4,7-anhKdo*f*			3.12	4.39	4.02	4.14	3.89	3.66, 3.60
		202.9	42.8	77.4	83.8	75.4	83.7	61.1
**Kdo’** 4,7-anhKdo*f*			3.09	4.51	4.12	4.08	3.74	3.67, 3.58
		203.2	38.8	75.8	80.2	74.7	84.8	61.8
**A** 4-α-Glc*p*N	5.48	3.33	3.94	3.63	3.74	3.78, 3.73		
	97.3	54.5	70.3	74.9	71.9	60.5		
**B** 2,7-l-α-d-Hep*p*	5.39	3.85	3.91	3.84	3.45	4.17	3.70	
	99.7	79.8	70.4	66.5	72.1	67.9	70.3	
**C**l-α- d-Hep*p*	5.27	3.96	3.74	3.81	3.54	3.94	3.62	
	101.2	70.1	70.5	65.9	72.5	68.5	62.7	
**D** α-Gal*p*NA	5.17	3.47	4.04	4.16	4.33			
	94.4	50.5	66.7	69.7	72.3	175.1		
**E** α-Glc*p*N	5.12	3.28	3.86	3.44	3.70	3.78, 3.72		
	96.0	54.1	69.8	69.4	72.2	60.2		
**F** 3,4-l-α- d-Hep*p*	5.07	3.90	3.84	4.23	3.53	3.93	3.67, 3.65	
	97.8	73.3	76.7	71.7	71.4	68.9	62.7	
**H** α-Glc*p*A	4.99	3.52	3.68	3.39	4.03			
	101.0	71.9	71.6	72.3	73.8	176.6		
**I ^b^** α-Glc*p*A	4.95	3.52	3.67	3.38	4.01			
	101.2	72.1	71.6	72.2	73.9	176.7		
**J** 4,6-β-Glc*p*	4.40	3.27	3.55	3.44	3.66	3.76, 3.89		
	101.4	73.4	76.4	79.0	73.0	67.3		

^a^ Spectra were obtained for ^2^H_2_O solutions at 25 °C. Acetone (δ_H_/δ_C_ 2.225/31.05 ppm) was used as internal reference ^b^ residue **I** is a variant of residue **H** in a different chemical environment. For comparison see Figure S1 and Table S1 of the supplementary data.

## Supplementary Materials

Supplementary materials can be found at https://www.mdpi.com/1422-0067/21/17/6433/s1.

## Abbreviations

LPS	Lipopolysaccharide
O-PS	O-specific polysaccharide
OS	Oligosaccharide
NMR	Nuclear Magnetic Resonance
COSY	Correlation spectroscopy
TOCSY	Total correlation spectroscopy
NOESY	Nuclear Overhauser Effect spectroscopy
HSQC	Heteronuclear Single Quantum Coherence
DEPT	Distortionless Enhancement by polarization transfer
HMBC	Heteronuclear Multiple Bond Correlation
GC	Gas chromatography
MS	Mass spectrometry
MALDI-TOF	Matrix-assisted laser desorption ionization time-of-flight
SDS	Sodium dodecyl sulfate
