# Supplementary material for: Bordetella holmesii Lipopolysaccharide Hide and Seek Game with Pertussis: Structural Analysis of the O-Specific Polysaccharide and the Core Oligosaccharide of the Type Strain ATCC 51541
Source: Int J Mol Sci. 2020 Sep 3;21(17):6433. doi: 10.3390/ijms21176433 (PMC7504554; doi:10.3390/ijms21176433)
Supplement: Supplementary file 1 [file ijms-21-06433-s001.pdf]

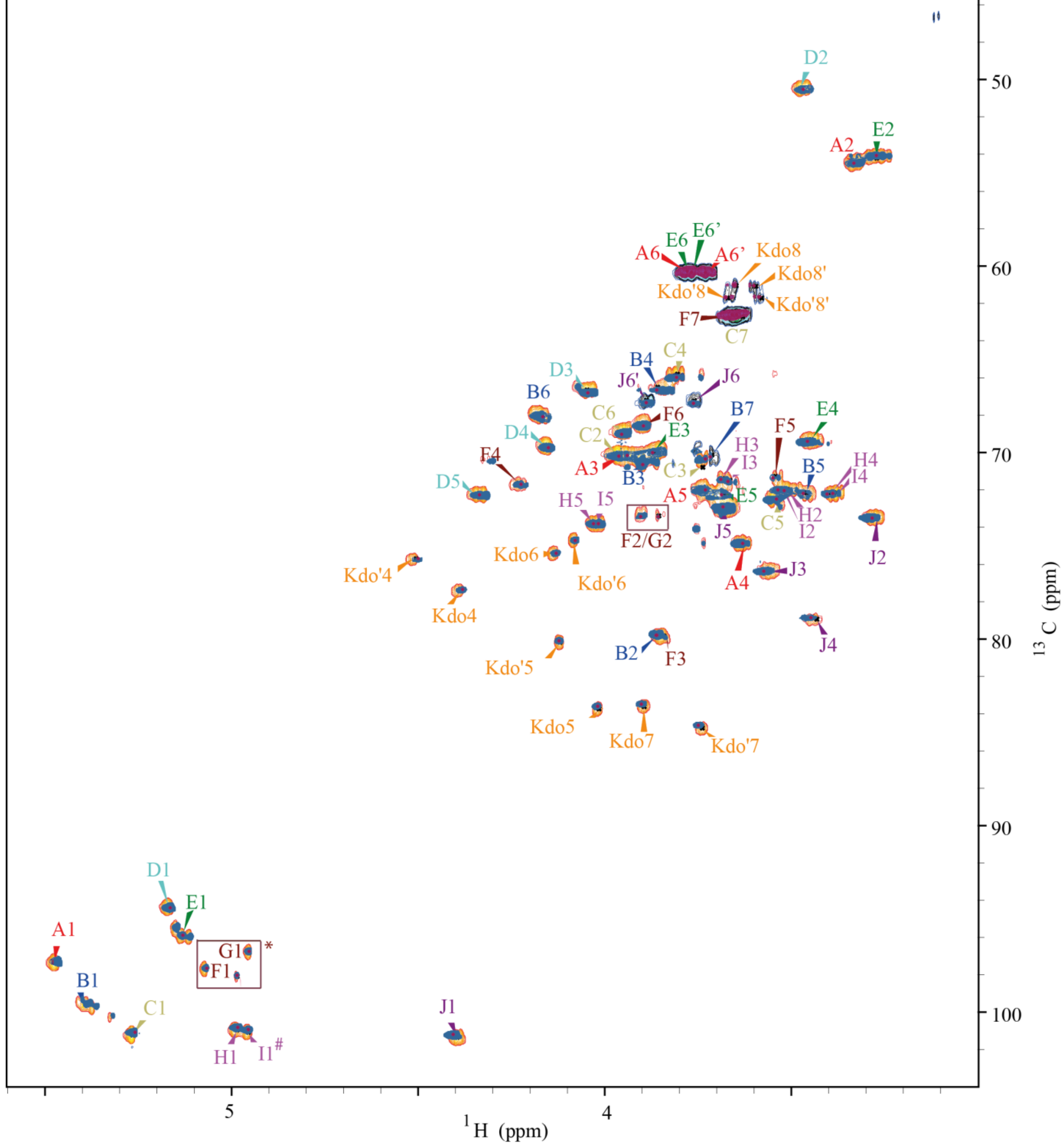

Figure S1. ^{1}H ^{13}C NMR HSQC-DEPT spectra of the OS of *B. holmesii* ATCC 51541 and *B. pertussis* 606 [overlay spectrum]; *Resonances placed in frames indicate variants of the residue F →3,4)-L-α-D-Hep*p*-(1→ in different environment; The remaining resonances of the initially identified residue G (δ_{H1}/δ_{C1} 4.95/96.9) and (δ_{H2}/δ_{C2} 3.85/73.4) were not resolved from those of residue F.

Table S1. ^{1}H ^{13}C NMR chemical shifts of the core oligosaccharides of *B. pertussis 606* and *B. holmesii* ATCC 51541 *

Residue	Strain	Chemical Shifts (ppm)							
		**H-1**	**H-2**	**H-3**	**H-4**	**H-5**	**H-6, H-6′**	**H-7**	**H-8, H-8′**
		**C-1**	**C-2**	**C-3**	**C-4**	**C-5**	**C-6**	**C-7**	**C-8**
**Kdo** →4,7)-anhKdo*f*	Bp606			3.12	4.38	4.02	4.13	3.89	3.66, 3.60
			203.1	42.9	77.4	83.8	75.4	83.6	61.1
	Bhol			3.12	4.39	4.02	4.14	3.89	3.66, 3.60
			202.9	42.8	77.4	83.8	75.4	83.7	61.1
**Kdo′** →4,7)-anhKdo*f*	Bp606			3.11	4.50	4.12	4.08	3.74	3.67, 3.58
			203.3	39.1	75.7	80.2	74.7	84.8	61.8
	Bhol			3.09	4.51	4.12	4.08	3.74	3.67, 3.58
			203.2	38.8	75.8	80.2	74.7	84.8	61.8
**A** →4)-α-Glc*p*N-(1→	Bp606	5.47	3.31	3.94	3.63	3.73	3.78, 3.73		
		97.4	54.5	70.3	74.9	71.9	60.5		
	Bhol	5.48	3.33	3.94	3.63	3.74	3.78	3.73	
		97.3	54.5	70.3	74.9	71.9	60.5		
**B** →2,7)-L-α-D-Hep*p*-(1→	Bp606	5.38	3.86	3.90	3.84	3.46	4.18	3.70	
		99.7	79.9	70.5	66.6	72.2	68.0	70.3	
	Bhol	5.39	3.85	3.91	3.84	3.45	4.17	3.70	
		99.7	79.8	70.4	66.5	72.1	67.9	70.3	
**C** L-α-D-Hep*p*-(1→	Bp606	5.27	3.96	3.64	3.78	3.55	3.90	3.65, 3.62	
		101.2	70.1	70.3	65.8	72.5	68.5	62.5	
	Bhol	5.27	3.96	3.74	3.81	3.54	3.94	3.62	
		101.2	70.1	70.5	65.9	72.5	68.5	62.7	
**D** α-Gal*p*NA-(1→	Bp606	5.16	3.44	4.03	4.15	4.33			
		94.5	50.5	66.7	69.7	72.3	175.1		
	Bhol	5.17	3.47	4.04	4.16	4.33			
		94.4	50.5	66.7	69.7	72.3	175.1		
**E** α-Glc*p*N-(1→	Bp606	5.12	3.28	3.85	3.45	3.70	3.78, 3.72		
		96.0	54.1	69.8	69.4	72.2	60.2		
	Bhol	5.12	3.28	3.86	3.44	3.70	3.78, 3.72		
		96.0	54.1	69.8	69.4	72.2	60.2		
**F** →3,4)-L-α-D-Hep*p*-(1→	Bp606	5.07	3.90	3.84	4.23	3.54	3.95	3.67, 3.65	
		97.7	73.5	79.9	71.7	71.3	68.9	62.7	
	Bhol	5.07	3.90	3.84	4.23	3.53	3.93	3.67, 3.65	
		97.8	73.3	76.7	71.7	71.4	68.9	62.7	
**H** α-Glc*p*A-(1→	Bp606	4.97	3.52	3.69	3.39	4.04			
		101.0	71.9	71.6	72.3	73.8	176.6		
	Bhol	4.99	3.52	3.68	3.39	4.03			
		101.0	71.9	71.6	72.3	73.8	176.6		
**I*** α-Glc*p*A-(1→	Bp606	4.96	3.51	3.67	3.37	4.01			
		101.1	72.1	71.5	72.2	73.8	176.7		
	Bhol	4.95	3.52	3.67	3.38	4.01			
		101.2	72.1	71.6	72.2	73.9	176.7		

Residue	Strain	Chemical Shifts (ppm)							
		H-1	H-2	H-3	H-4	H-5	H-6, H-6′	H-7	H-8, H-8′
		C-1	C-2	C-3	C-4	C-5	C-6	C-7	C-8
**J** →4,6)-β-Glc*p*-(1→	Bp606	4.39	3.27	3.56	3.44	3.67	3.76, 3.89		
		101.4	73.4	76.4	78.9	72.9	67.2		
	Bhol	4.40	3.27	3.55	3.44	3.66	3.76, 3.89		
		101.4	73.4	76.4	79.0	73.0	67.3		

* The additional CH_2- signals at δ_{H1}/δ_{C1} (3.13/40.5 ppm), $\delta_{H1'}/\delta_{C1'}$ (3.18/39.8 ppm) and δ_{H2}/δ_{C2} (3.9/60.2 ppm), $\delta_{H2'}/\delta_{C2'}$ (4.09/62.0 ppm) for the *B. pertussis* 606 OS and at δ_{H1}/δ_{C1} (3.17/40.0 ppm) and δ_{H2}/δ_{C2} (4.07/62.2 ppm) for the *B. holmesii* OS V indicate the presence of phosphoethanolamine in the intact core oligosaccharides of these strains. P-EtN correlations were not resolved and the substitution position is only tentative.

Table S2. Selected inter-residue NOE and $^{3}J_{H,C}$-connectivities from the anomeric atoms of the core oligosaccharide of *B. pertussis* 606

Residue	Atom H-1/C-1 Connectivities to			Inter-Residue
	(ppm)	δC	δH	Atom/Residue
**A** →4)-α-Glc*p*N-(1→	97.4/5.47	78.9	3.44	C-4, H-4 of **J**
**B** →2,7)-L-α-D-Hep*p*-(1→	99.7/5.38	79.9	3.84	C-3, H-3 of **F**
**C** L-α-D-Hep*p*-(1→	101.2/5.27	74.9	3.63	C-6, H-6/6′ of **A**
**D** α-Gal*p*NA-(1→	94.5/5.15	67.2	3.76/3.89	C-6, H-6/6′ of **J**
**E** α-Glc*p*N-(1→	96.0/5.12	70.3	3.70	C-7, H-7 of **B**
**F**→3,4)-L-α-D-Hep*p*-(1→	97.7/5.07	83.7	4.02	C-5, H-5 of **Kdo**
**H** α-Glc*p*A-(1→	101.0/4.97	79.9	3.86	C-2, H-2 of **B**
**I** α-Glc*p*A-(1→	101.1/4.96	79.9	3.85	C-2, H-2 of **B**
**J**→4,6)-β-Glc*p*-(1→	101.4/4.39	71.7	4.23	C-4, H-4 of **F**
